# Isolation and evolutionary analysis of feline panleukopenia virus strains FPV-BJ-J2 and FPV-BJ-J3 (T440A, N564S, A568G) in Beijing, China

**DOI:** 10.3389/fvets.2026.1745551

**Published:** 2026-01-27

**Authors:** Xia Su, Hongzhuan Zhou, Wenqian Jiang, Fuzhou Xu, Bing Xiao, Jin Zhang, Qi Qi, Bing Yang

**Affiliations:** Beijing Key Laboratory for Prevention and Control of Infectious Diseases in Livestock and Poultry, Institute of Animal Husbandry and Veterinary Medicine, Beijing Academy of Agriculture and Forestry Sciences, Beijing, China

**Keywords:** evolutionary trend, feline panleukopenia virus, mutation site, T440A, VP2 protein

## Abstract

Feline panleukopenia virus (FPV) is a single-stranded linear DNA virus with high lethality, whose VP2 protein determines viral host range and antigenicity. Substitutions at several key VP2 residues are closely associated with enhanced virulence and immune evasion. However, their patterns and temporal evolutionary dynamics remain poorly characterized. In this study, we analyzed global FPV VP2 gene sequences that were retrieved from the NCBI database and seven FPV VP2 sequences newly isolated by our laboratory during 2024–2025. Multiple sequence alignment was performed using MAFFT, and phylogenetic trees were constructed with IQ-TREE. Meanwhile, mutation characteristics were further analyzed using Shannon entropy. The results revealed that VP2 hypervariable regions were mainly concentrated in nt 111–411, nt 477–1,038, and nt 1,500–1752, with the highest entropy peak at nt 271 (aa 91), which corresponds to the A91S substitution. Temporal dynamics analysis revealed that the frequency of the A91S substitution has markedly increased since 2017, suggesting ongoing positive selection. The high-frequency I101T substitution has stabilized, suggesting an adaptive equilibrium. Conversely, the substitution frequency at residue 232 has gradually declined over time. Notably, this study for the first time identified a T440A substitution in the newly isolated FPV-BJ-J2 and FPV-BJ-J3 strains. This 440A site, which is located on the viral capsid surface, co-occurs with N564S and A568G, exhibiting the characteristic substitution combination of CPV-2c, which may be associated with enhanced immune evasion of FPV. Overall, this study systematically reveals the temporal evolutionary characteristics of key VP2 residues, providing important theoretical insights for FPV molecular epidemiology and vaccine strain optimization.

## 1 Introduction

FPV (Feline Panleukopenia Virus) belongs to the genus *Protoparvovirus* within the family *Parvoviridae* (1) and is the primary pathogen responsible for feline panleukopenia (FPL) (2). This virus exhibits an extremely high infection and mortality rate in domestic cats. The mortality can reach up to 80% in kittens (3, 4). FPV is mainly transmitted via the oral and nasal routes and targets rapidly dividing cells, such as intestinal crypt epithelial cells, bone marrow hematopoietic cells, and lymphoid tissue cells, leading to acute vomiting, diarrhea, and marked leukopenia (2, 5). Members of the *Felidae*, *Mustelidae*, and *Procyonidae* families are all susceptible to FPV, with an overall mortality rate ranging from approximately 50 to 80% (2). In addition, cats can also be infected by variant strains of canine parvovirus (CPV), including CPV-2a, CPV-2b, and CPV-2c, which cause clinical symptoms that are difficult to distinguish from feline panleukopenia (FPL). Notably, multiple or mixed infections may facilitate genetic recombination between viruses, thereby accelerating the ongoing evolution of parvovirus populations (6, 7).

FPV is a non-enveloped, single-stranded linear DNA (ssDNA) virus with a viral particle diameter of approximately 20–25 nm and a genome length of ~5.2 kb, flanked by hairpin-shaped terminal palindromic sequences (8, 9). Its genome contains two major open reading frames (ORFs), which encode non-structural proteins (NS1/NS2) and structural proteins (VP1/VP2), respectively. VP1 and VP2 are produced via alternative splicing, the full VP2 sequence contained within VP1 (10). Constituting approximately 90% of the viral capsid, VP2 is composed of 584 amino acids and represents the most immunogenic protein, playing a key role in determining host tropism and antigenicity (11, 12). FPV shares high genomic homology with canine parvovirus type 2 (CPV-2), yet differences of only three to four amino acid residues on the VP2 surface can result in altered host specificity (13). Wang et al. (14) reported that global FPV strains can be classified into three major genetic groups (FPV-G1, FPV-G2, and FPV-G3), with FPV-G3 further divided into multiple sublineages (G3A–G3H) and emerging as the predominant circulating lineage worldwide over the past two decades.

Given that substitutions in the capsid protein VP2 may affect viral antigenicity, pathogenicity, and host adaptation, this study systematically analyzed the genetic diversity and evolutionary characteristics of key amino acid residues of FPV VP2. The analysis was based on sequences of FPV strains isolated from clinical diarrhea cases in Beijing, combined with publicly available global FPV VP2 sequences from the NCBI database. Special attention was given to substitution patterns associated with host adaptation and immune evasion, as well as their temporal evolutionary trends, providing molecular-level theoretical insights for parvovirus vaccine optimization and epidemiological control.

## 2 Materials and methods

### 2.1 Clinical samples

**Figure 1:**
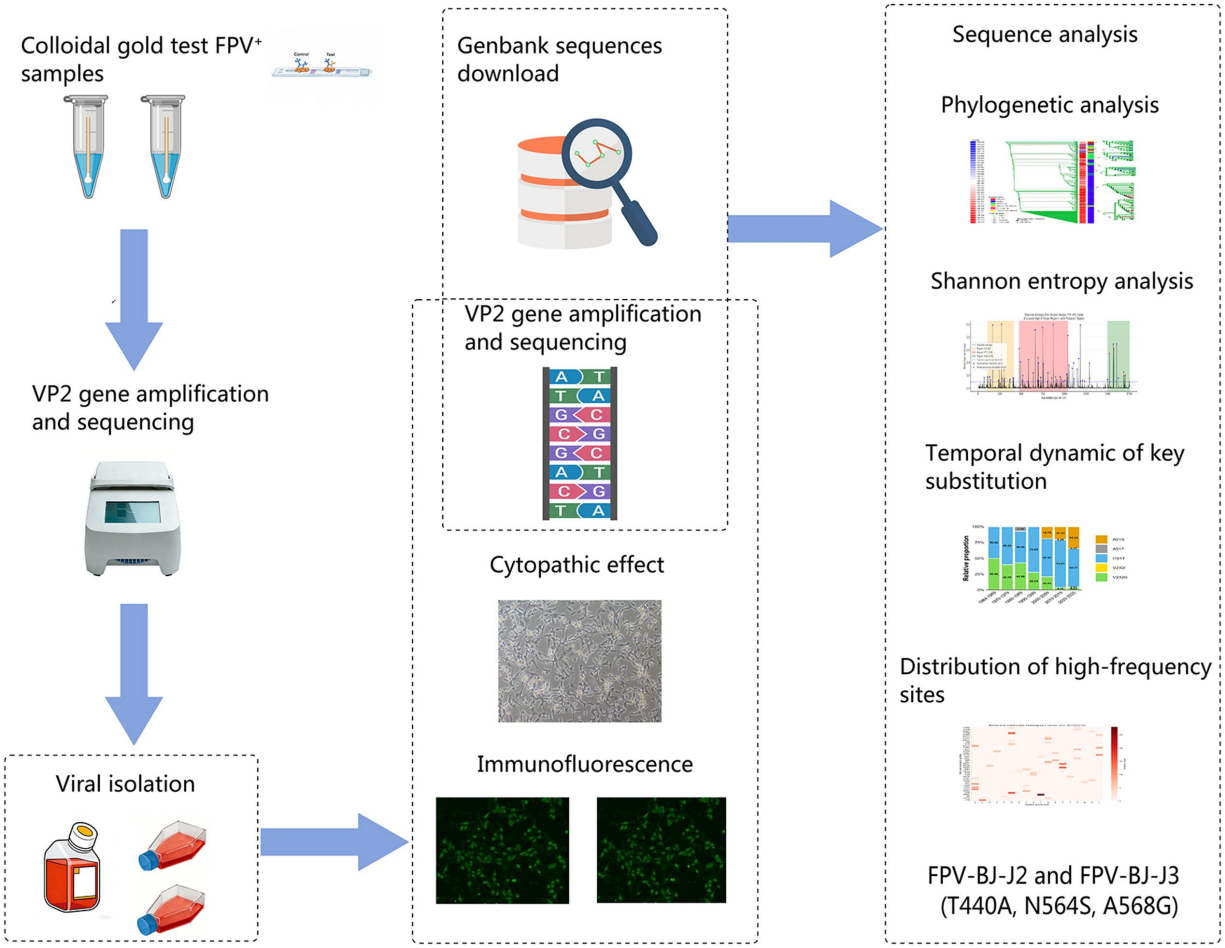
Workflow of the study. The diagram illustrates the key steps from clinical sample collection, virus isolation, and VP2 sequence dataset construction to subsequent bioinformatics analyses, providing an overview of the experimental and analytical procedures.

Clinical samples were obtained from diseased cats at pet hospitals in Beijing during 2024–2025. The key steps of sample collection, virus isolation, sequence dataset construction, and subsequent bioinformatics analyses are summarized in Figure 1. These cats presented with diarrhea and vomiting, and hematological examination revealed a significant decrease in white blood cell counts, with clinical diagnosis suspected as FPV infection. Fecal samples were collected using sterile swabs, suspended in sterile physiological saline, and subjected to three cycles of freeze–thaw to lyse viral particles. The swab suspensions were then expressed to obtain the supernatant, which was centrifuged at 10,000 × g for 5 min. The resulting supernatant was mixed with the appropriate ratio of penicillin–streptomycin solution, filtered through a 0.22 μm microporous filter, and stored at −80 °C for further use.

### 2.2 Reagents

RPMI 1640 medium, fetal bovine serum (FBS), Penicillin (100 U/mL) and streptomycin (0.1 mg/mL) were purchased from Thermo Fisher Scientific (Thermo Fisher Scientific, Waltham, MA, United States). DNA extraction kits were purchased from Cwbio (CWBIO, Beijing, China). FastPfu DNA polymerase, 5 × Fast Pfu buffer, dNTPs, the pEASY-Blunt Zero cloning kit, and T1 competent cells were obtained from TransGen Biotech (TransGen Biotech, Beijing, China). 0.22 μm syringe filters were purchased from Merck Millipore (Merck Millipore, Burlington, MA, United States). Parvovirus antibody (CPV1-2A1, sc-57961) was obtained from Santa Cruz (Santa Cruz, Dallas, TX, USA). All primers were synthesized by Sangon Biotech (Sangon Biotech, Shanghai, China).

### 2.3 VP2 gene cloning and sequencing

Fecal samples were diluted 10-fold with DMEM medium (Thermo Fisher Scientific, Waltham, MA, United States), and viral genomic DNA was extracted following the instructions of the CWBIO viral DNA extraction kit (CWBIO, Beijing, China). PCR amplification of the VP2 gene was performed using specific primers (Sangon Biotech, Shanghai, China) (Table 1). PCR was conducted to amplify each fragment using FastPfu DNA polymerase (TransGen Biotech, Beijing, China), including 4 μL 5 × FastPfu Buffer, 2 μL dNTPs (2.5 mM), 0.8 μL each primer (10 μM), 0.4 μL FastPfu Polymerase, 1 μL (10 ng) template DNA, with molecular biology grade water for a final volume of 20 μL. Amplification is carried out under the following conditions: initial denaturation at 95 °C for 2 min, 30 cycles of denaturation at 95 °C for 30 s, annealing at 54 °C for 30 s, and extension at 72 °C for 1 min, and an additional final extension at 72 °C for 5 min. The amplified products were analyzed on a 10 g/L agarose gel and purified. The purified products were cloned into the pEASY-Blunt Zero vector (TransGen Biotech, Beijing, China) and ligated overnight at 16 °C. The ligation products were then transformed into T1 competent cells (TransGen Biotech, Beijing, China) and plated on LB agar containing ampicillin for 12 h. Single colonies were picked and inoculated into liquid LB medium, cultured at 37 °C with shaking at 180 rpm for 14 h, and positive clones were verified by PCR. Positive clones were sent to Sangon Biotech (Sangon Biotech, Shanghai, China) for Sanger dideoxy sequencing, and the obtained VP2 sequences were subsequently assembled individually using SnapGene version 6.0.2.

**Table 1:** Primers used for amplification of the full-length FPV VP2 gene.

Name	Sequence	Position	Length
FPV-VP2-F1	TCAATCTTGCAAAAAAAAAAAAAGCCGGTG	−62– −33	987
FPV-VP2-R1	GAACTCCTATATCACCAAAGTTAGTAGCTCC	895–925	987
FPV-VP2-F2	CTCAAGCAGACTTGTACATTT	192–212	1,114
FPV-VP2-R2	TGGATCTGTTGGTAGCAATAC	1,285–1,305	1,114
FPV-VP2-F3	GATATGCATTTGGTAGACAACATGGTCAAAA	1,130–1,160	786
FPV-VP2-R3	CCTTCTAAATCCTATATCAAATACAAGTACAATATTTCTATGCTG	1871–1915	786

### 2.4 Virus isolation and culture

The feline kidney cell line F81 (preserved at the Laboratory of Beijing Academy of Agriculture and Forestry Sciences) were maintained in RPMI 1640 complete medium supplemented with 10% FBS (Thermo Fisher Scientific, Waltham, MA, United States), 100 U/mL penicillin, and 0.1 mg/mL streptomycin (Thermo Fisher Scientific, Waltham, MA, United States) at 37 °C in a humidified incubator containing 5% CO₂. Virus isolation was performed using a synchronous inoculation method. During cell passaging, the processed viral samples were inoculated into F81 cells at infection ratios of 1:5, 1:10, and 1:15 (sample:cell culture medium). After inoculation, the cells were incubated at 37 °C with 5% CO₂ and monitored for cytopathic effects (CPE) using a Nikon ECLIPSE TS 100 microscope. Typical CPE included cell rounding, shrinkage, detachment from the culture surface, and progressive cell lysis. Representative images were captured with a Nikon DIGITAL SIGHT DS-U3 digital camera (Nikon Corporation, Tokyo, Japan). When approximately 80% of the cells exhibited characteristic CPE, the cultures were harvested and subjected to three freeze–thaw cycles. Cells and supernatants were then collected and stored at −80 °C for further use.

### 2.5 Immunofluorescence assay

F81 cells were seeded in 96-well plates and cultured at 37 °C with 5% CO₂ for approximately 24 h until cells adhered and reached appropriate confluence. Uninfected cells were used as negative controls. After removing the culture medium, cells were washed three times with PBS and fixed with 50 μL of 4% paraformaldehyde (Sigma-Aldrich, Saint Louis, MO, United States) at room temperature for 45 min. Following fixation, the fixative was removed, and cells were permeabilized with 50 μL of 0.3% Triton X-100 (Sigma-Aldrich, Saint Louis, MO, United States) for 1 h, then washed three times with PBS. Cells were then incubated with parvovirus antibody against FPV VP2 protein (CPV1-2A1, sc-57961, Santa Cruz, Dallas, TX, United States), 50 μL per well, at 37 °C in the dark for 1.5 h, followed by three washes with PBS. Subsequently, FITC-conjugated secondary antibody (1:200, Sigma-Aldrich, Saint Louis, MO, USA), 50 μL per well, was added and incubated at 37 °C in the dark for 45 min, followed by three washes with PBS. Fluorescence was observed using an Axio Observer. Z1 inverted fluorescence microscope (Zeiss, Germany) at 10 × magnification.

### 2.6 Sequence acquisition and alignment

As of September 10, 2025, a total of 1,741 sequences related to Feline Panleukopenia Virus were retrieved from the NCBI Nucleotide database using the keyword “Feline Panleukopenia Virus”. Sequence information, including strain name, year and region, was organized using R 4.2.3 (R Foundation for Statistical Computing, Vienna, Austria) and relevant packages (see Supplementary Table S1), with missing information manually verified and supplemented. Sequences with incomplete metadata or lacking full-length VP2 were subsequently removed.

### 2.7 Phylogenetic analysis and construction of maximum likelihood trees

Multiple sequence alignment of 708 full-length FPV VP2 gene sequences, including seven sequences obtained in this study and 701 sequences from GenBank (Supplementary Table S1), was performed using MAFFT v7.037 (15) with the L-INS-i algorithm and default parameters. Subsequently, a maximum likelihood phylogenetic tree was constructed using IQ-TREE v2.4.0 (16). The best-fit substitution model, TIM2 + F + R3, was selected by ModelFinder (17) according to the Bayesian Information Criterion (BIC). Canine parvovirus type 2 (CPV-2) was used as the outgroup, and branch support was assessed with 5,000 bootstrap replicates to determine the root of the phylogenetic tree. The resulting phylogenetic tree was visualized using the Chiplot online tool (18).

### 2.8 Analysis of sequence mutation site entropy

The VP2 coding region sequences obtained from MAFFT alignment were imported into R 4.2.3 (R Foundation for Statistical Computing, Vienna, Austria) for further analysis. The nucleotide mutation frequency and Shannon entropy values at each site were then calculated using the bio3d (19) package in R. The entropy values were calculated using the formula H = −∑p_i_log_2_p_i_ (20), where pi represents the frequency of the i-th base at that site, with a high-variation threshold set as H ≥ *μ* + 2σ (mean plus two standard deviations). The resulting entropy matrix was organized with dplyr and visualized with ggplot2 (21).

### 2.9 Statistical analysis and heatmap visualization of amino acid substitution trends

VP2 sequences obtained from MAFFT v7.037 (15) alignment were imported into R 4.2.3 (R: The R Project for Statistical Computing) for substitution frequency calculation and visualization. Data processing was primarily performed using the R packages tidyverse (22), stringr (23) and readxl (24), while figures were generated using ggplot2 (21).

## 3 Results

### 3.1 Sequence determination and isolation of FPV strains

**Figure 2:**
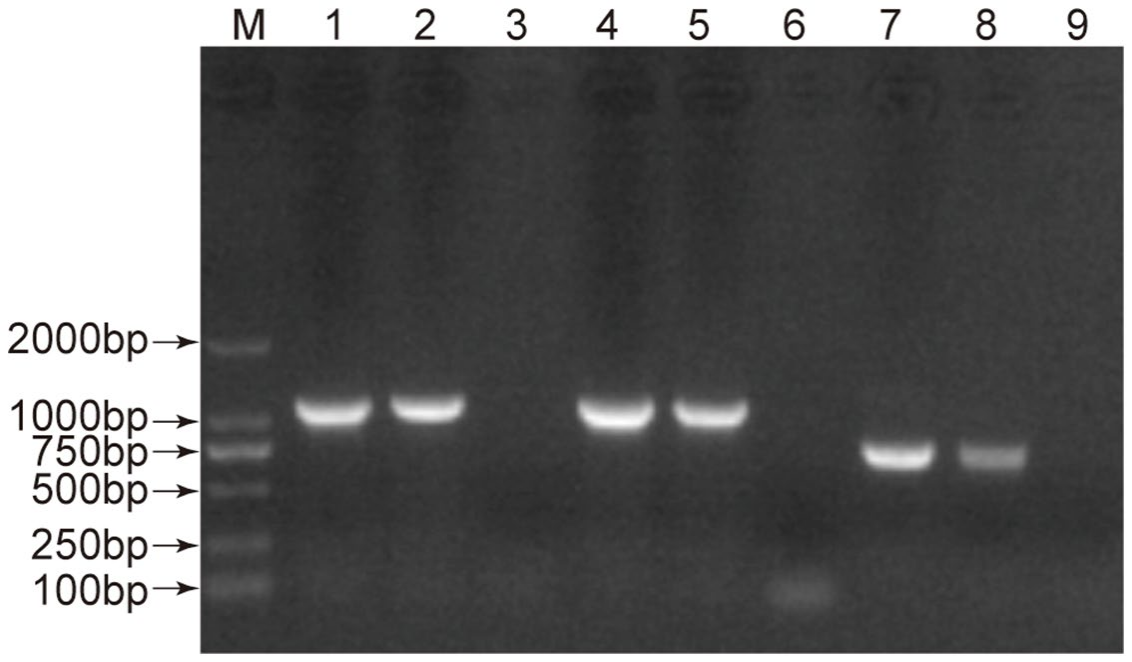
Identification of PCR amplification products of the FPV VP2 gene. M: DNA marker; Lanes 1, 2: first amplified fragment (1,114 bp) of FPV-BJ-J2 and FPV-BJ-J3; Lane 3: negative control; Lanes 4, 5: second amplified fragment (987 bp) of FPV-BJ-J2 and FPV-BJ-J3; Lane 6: negative control; Lanes 7, 8: third amplified fragment (786 bp) of FPV-BJ-J2 and FPV-BJ-J3; Lane 9: negative control.

Initially, the clinical samples were preliminarily assessed as positive based on clinical symptoms and colloidal gold testing. Then, fecal samples were subjected to PCR amplification using three overlapping fragments to obtain the complete VP2 gene. As shown in Figure 2, three amplicons of 1,114 bp, 987 bp, and 786 bp were successfully generated from the FPV-BJ-J2 and FPV-BJ-J3 samples, consistent with the expected fragment sizes. Using the same strategy, a total of seven high-quality full-length VP2 sequences (1755 bp) were obtained through amplification, cloning, sequencing, and assembly. These samples were subjected to virus isolation and, after sequence confirmation, all strain sequence information (FPV-BJ-J1, FPV-BJ-J2, FPV-BJ-J3, FPV-BJ-J4, FPV-BJ-Y1, FPV-BJ-KBwy, and FPV-BJ-KBbb) was uploaded to GenBank (Table 2). BLAST analysis revealed that these sequences shared 99.54–100% nucleotide identity with Top 2 GenBank hits (Table 2).

**Table 2:** BLAST analysis of sequence homology of the isolated strains (Top 2).

GenBank number	Strains	Collection date	GenBank Number of Hit 1 (Percent Identity)	GenBank Number of Hit 2 (Percent Identity)
PX496583	FPV-BJ-J1	20-Nov-2024	PP619435.1 (99.94%)	PQ560987.1 (99.89%)
PX496584	FPV-BJ-J2	20-Nov-2024	OK384314.1 (99.66%)	PP619435.1 (99.66%)
PX496585	FPV-BJ-J3	20-Nov-2024	OK384314.1 (99.54%)	PP619435.1 (99.54%)
PX496586	FPV-BJ-J4	20-Nov-2024	PP619435.1 (100%)	PQ560987.1 (99.94%)
PX496587	FPV-BJ-Y1	07-Jul-2025	OR921195.1 (99.83%)	OR727319.1 (99.83%)
PX496588	FPV-BJ-KBwy	13-Jan-2025	PP619435.1 (100%)	PQ560987.1 (99.94%)
PX496589	FPV-BJ-KBbb	13-Jan-2025	PP619435.1 (100%)	PQ560987.1 (99.94%)

### 3.2 Viral identification and immunofluorescence analysis

**Figure 3:**
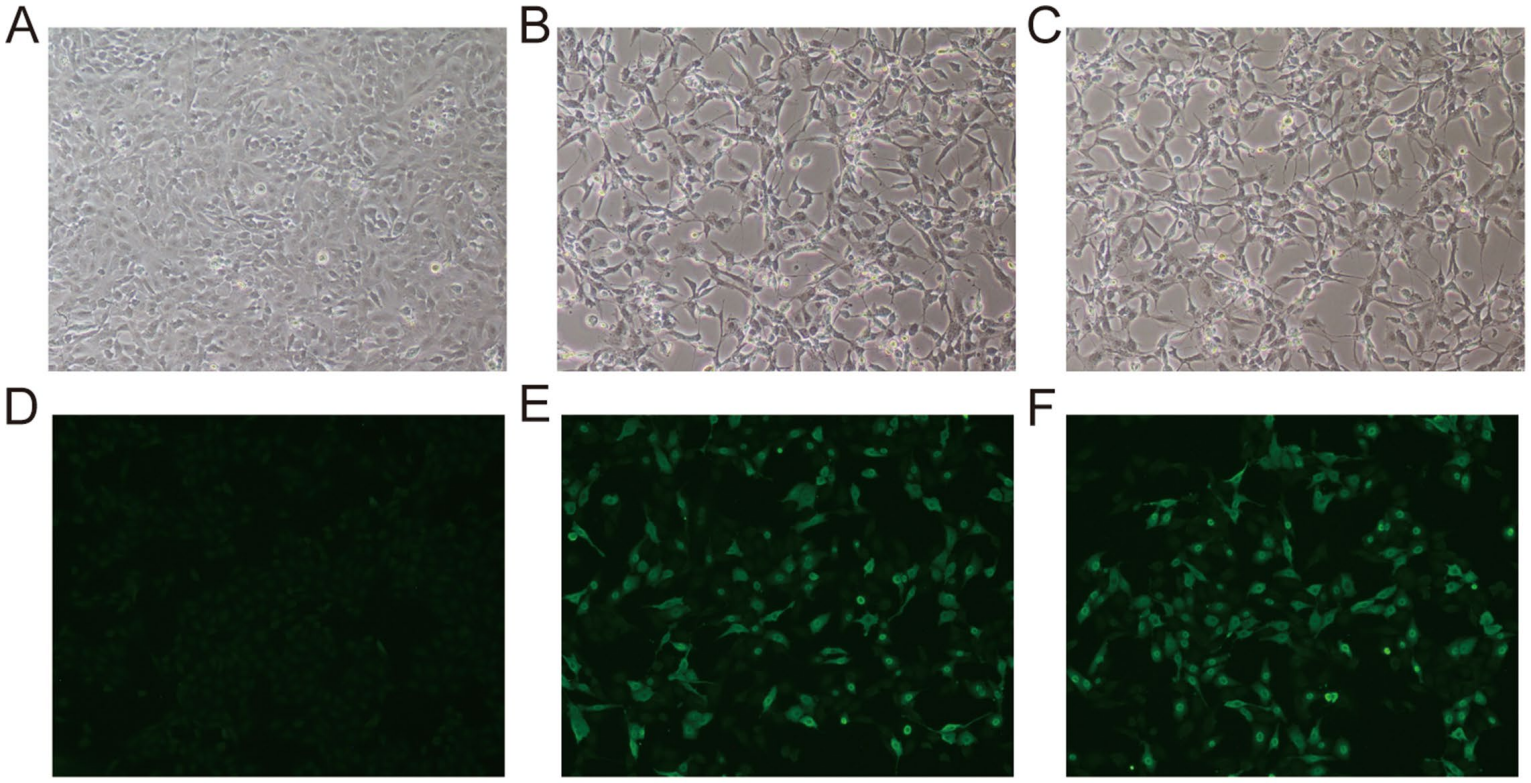
Cytopathic effects (CPE) and immunofluorescence assay of FPV-infected F81 cells. **(A)** Uninfected control F81 cells showing normal morphology without apparent cytopathic changes. **(B,C)** FPV-infected F81 cells (fifth passage, P5) displaying typical CPEs, including cell rounding, shrinkage, and detachment. **(D)** Uninfected control F81 cells showing no green fluorescence. **(E,F)** FPV-infected F81 cells (P5) exhibiting strong specific green fluorescence signals mainly localized in the cytoplasm, indicating expression of the VP2 protein and specific recognition by FPV antibodies.

Cytopathic effect (CPE) observation and immunofluorescence assay (IFA) were further performed to confirm the infectivity and specificity of the isolated virus. Virus were inoculated onto F81 cells, and typical CPEs were observed 48–72 h post-infection, characterized by cell rounding, shrinkage, detachment, and vacuolization (Figures 3B,C). After five serial blind passages (P5), the CPE became more evident and remained stable. To further verify the specificity of the isolated virus, an immunofluorescence assay was conducted using P5-infected cells. As shown in Figures 3E,F, strong green fluorescence signals were detected in infected cells, mainly localized in the cytoplasm, with some cells showing slight morphological alterations. In contrast, no fluorescence signal was observed in the uninfected control cells, which retained normal morphology (Figures 3A,D). These results indicate that the isolated virus can stably replicate in F81 cells and express the VP2 protein, which can be specifically recognized by FPV-specific antibodies.

### 3.3 Phylogenetic analysis

**Figure 4:**
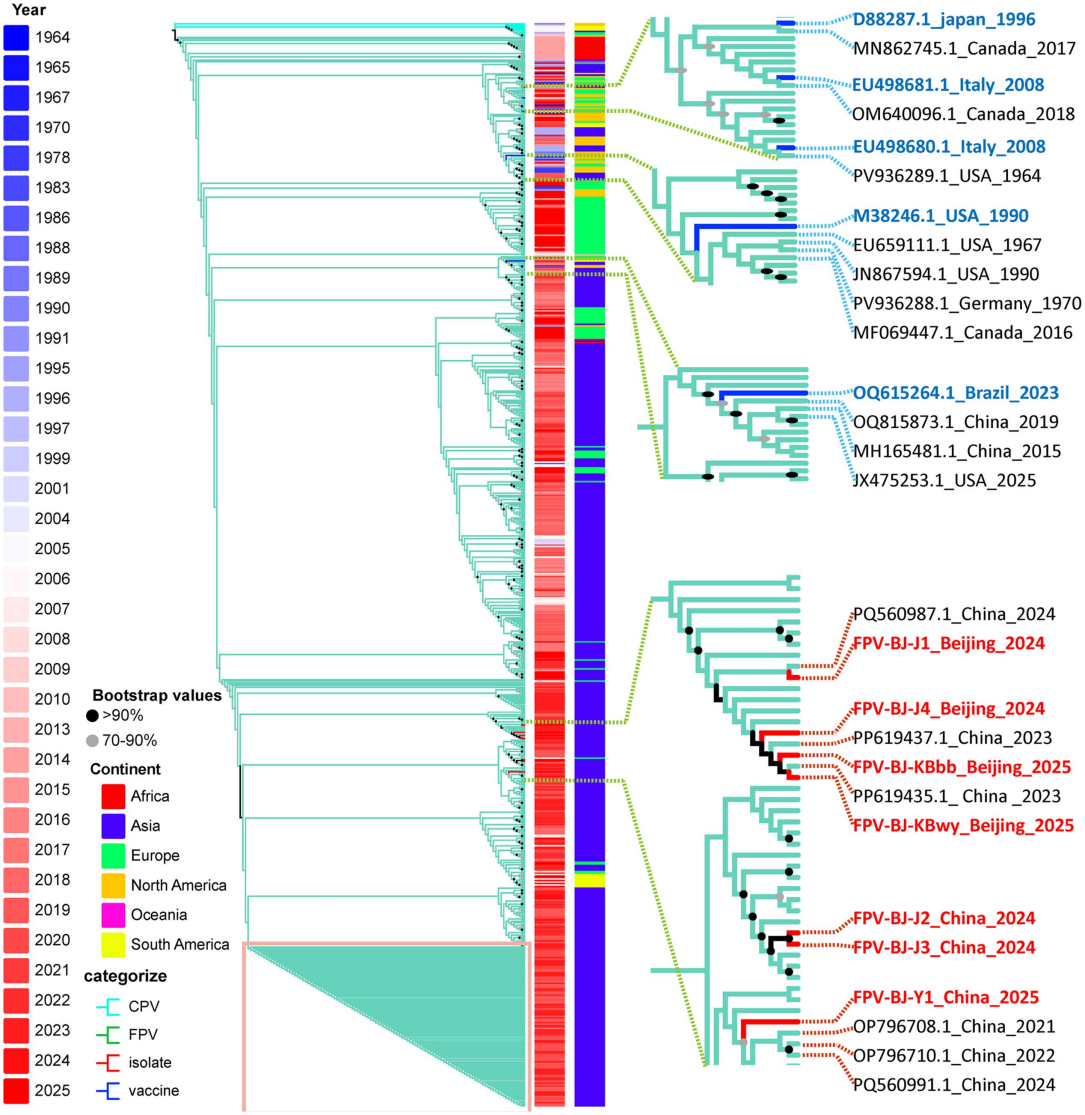
Maximum-likelihood phylogenetic tree of FPV/CPV based on complete VP2 sequences (TIM2 + F + R3 model, *n* = 717). Red labels on the right indicate FPV isolates identified in this study; blue labels represent reference vaccine strains from GenBank; and green labels denote CPV sequences. The heatmap on the right shows the geographic origins of the sequences, while the heatmap on the left indicates sampling years, revealing the evolutionary clustering patterns of FPV across different time periods. Branch colors correspond to distinct evolutionary clades, and the numbers shown on major branches represent bootstrap support values (≥70%).

On the basis of strain isolation, phylogenetic analysis of the VP2 gene was performed to determine their genetic relationships and evolutionary origins. A maximum-likelihood phylogenetic tree was constructed based on 708 FPV VP2 sequences (including the seven isolates obtained in this study), with nine CPV sequences used as the outgroup (Figure 4; Supplementary Table S1). ModelFinder identified TIM2 + F + R3 as the best-fit substitution model according to the Bayesian information criterion (BIC; log-likelihood = −10238.387). Branch support was evaluated using 5,000 ultrafast bootstrap replicates, and the topology convergence correlation coefficient reached 0.990, indicating a stable tree topology. The phylogenetic analysis revealed that FPV formed an independent and well-supported major clade. Notably, 106 sequences in the dataset shared 100% nucleotide identity, all originating from China (2014–2018) and clustered into a single monophyletic group (highlighted in pink in Figure 4), suggesting a high degree of genetic homogeneity and strong temporal clustering among these strains.

The seven FPV strains isolated in this study (FPV-BJ-J1, FPV-BJ-J2, FPV-BJ-J3, FPV-BJ-J4, FPV-BJ-Y1, FPV-BJ-KBwy, and FPV-BJ-KBbb) were located on distinct branches from the reference vaccine strains (D88287, EU49868, M38246, and OQ615264), indicating marked genetic divergence (Figure 4). This divergence was primarily associated with multiple amino acid substitutions in the N- and C-terminal regions of the VP2 gene, including key substitutions such as A91S and I101T, suggesting that the circulating FPV strains have formed different evolutionary lineages. Several FPV vaccine or candidate strains have been reported in China—such as HBX05, FP/15, PSY01, CS-2016, 0918, and RPVF0110—yet their complete gene sequences have not been publicly documented. Therefore, these strains were not included in the present phylogenetic analysis.

At the geographical level, FPV sequences from different regions exhibited a partially mixed distribution on the phylogenetic tree, yet regional clustering patterns were still evident. Sequences originating from Asia formed a relatively independent cluster (blue region in Figure 4), within which a few South American strains—such as the Argentine isolate EU018145.1—were also grouped. European sequences showed a broader distribution and overlapped with those from North America, Africa, and Asia (mixed red, yellow, blue, and green regions in Figure 4), suggesting a complex global transmission pattern and ongoing genetic exchange of FPV worldwide.

### 3.4 Entropy analysis of the VP2 gene nucleotide sequences

**Figure 5:**
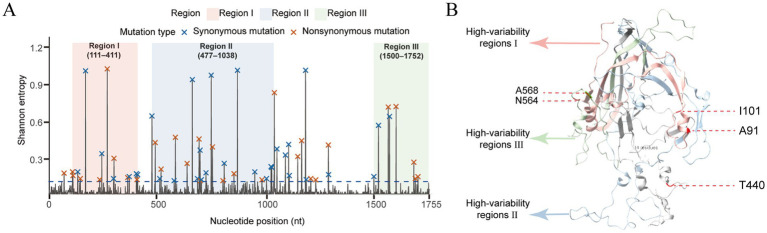
Shannon entropy analysis of the FPV VP2 coding region reveals three high-variability regions and the distribution of mutation types. **(A)** Shannon entropy analysis identified three high-variability regions within the FPV VP2 coding sequence and characterized the distribution of mutation types. The blue dashed line indicates the high-entropy threshold (0.1), and the background shaded regions—highlighted in red, blue, and green—correspond to the three focal high-variability regions (nt 111–411, 477–1,038, and 1,500–1752). Red dots represent nonsynonymous substitutions, while blue dots represent synonymous substitutions. **(B)** In the VP2 three-dimensional structure (PDB: 1C8E) (25), the core *β*-barrel is shown in gray; the first (aa 37–137), second (aa 159–347), and third (aa 500–584) hypervariable regions identified by Shannon entropy analysis are highlighted in windsor mist pink, blue, and green, respectively. A91, I101, T440, N564, A568 residues are marked in red.

To further elucidate the molecular variation characteristics and structural distribution patterns of the FPV VP2 gene, Shannon entropy analysis was performed on 708 full-length VP2 sequences to quantify the conservation and variability across different regions (Figures 5A,B; Supplementary Table S2). Based on the three-dimensional structure of the FPV capsid reported by Simpson et al. (25) (PDB ID: 1C8E) and Tsao et al. (11), the VP2 protein consists of a core β-barrel framework and four major surface loops (Loop 1–4), along with an N-terminal region located near the fivefold axis channel and an exposed C-terminal tail. The analysis revealed three major hypervariable regions in the VP2 protein: The first hypervariable region (nt 111–411, aa 37–137) is located at the N-terminus and adjacent to Loop 1, near the base of the fivefold axis (Figures 5A,B); The second hypervariable region (nt 477–1,038, aa 159–347) corresponds to the surface Loops 2–4, which form the spike-like protrusions around the threefold axis, and exhibits a high proportion of nonsynonymous substitutions (Figures 5A,B); The third hypervariable region (nt 1,500–1752, aa 500–584) lies in the exposed C-terminal region, near the interface between the threefold and twofold axes, encompassing known antigenic determinant clusters that include key residues such as 564 and 568 (highlighted by windsor mist pink, blue, and green shaded areas in Figures 5A,B). Overall, the sequence variation of VP2 is predominantly concentrated in the surface-exposed loop regions and the C-terminal area. The spatial distribution of these variable sites is highly consistent with the protein’s surface exposure and functional domain organization.

### 3.5 Analysis of the evolutionary trends of residues 91, 101, and 232

**Figure 6:**
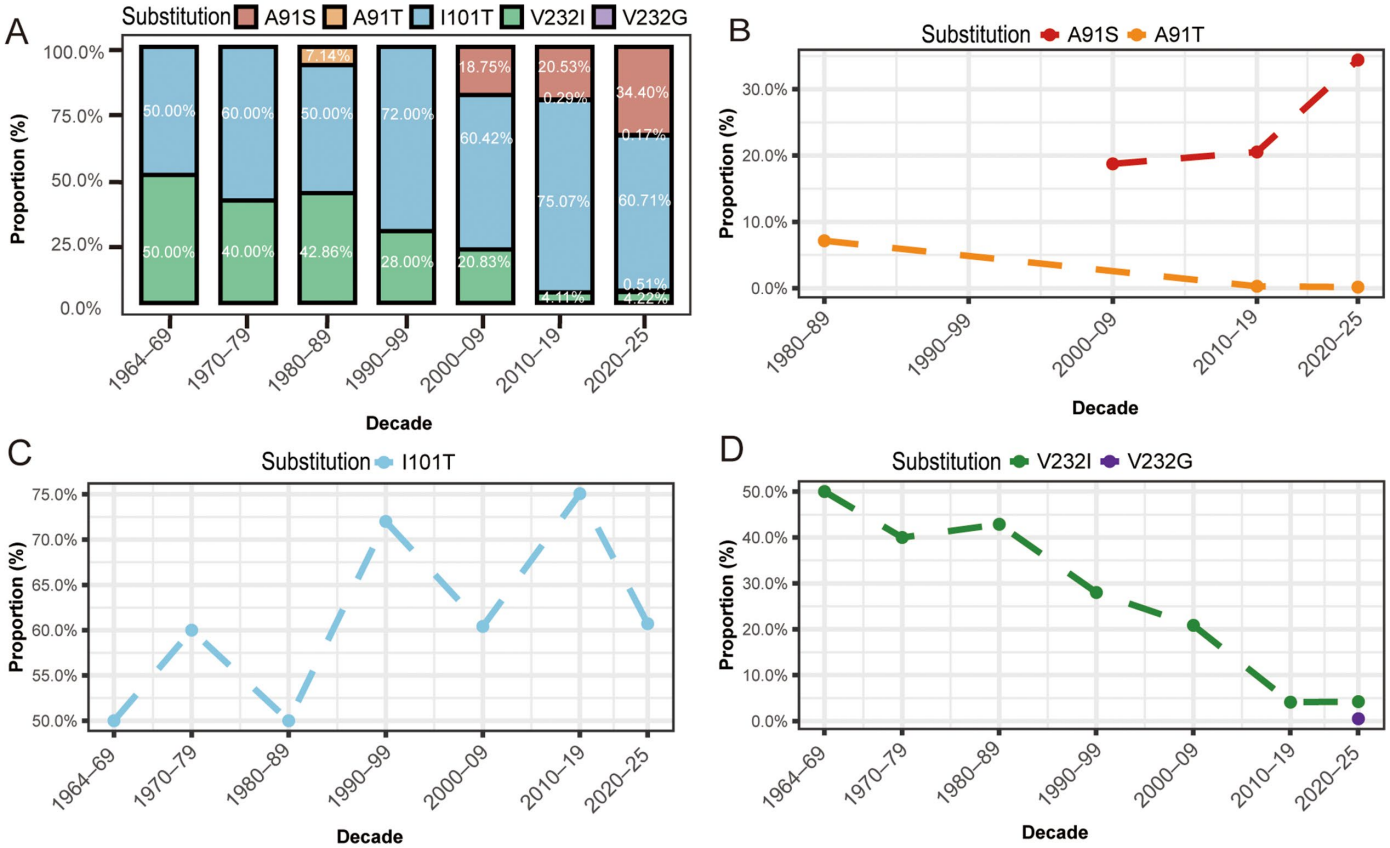
Temporal evolutionary trends of key VP2 residue substitutions in FPV. **(A)** Temporal substitution frequency changes of residues 91, 101, and 232 across different decades; **(B)** Decadal substitution frequency of residue 91 showing positive selection toward serine substitution; **(C)** Decadal substitution frequency of residue 101 maintaining high frequency with minor fluctuation; **(D)** Decadal substitution frequency of residue 232 and emergence of the novel V232G substitution, indicating a gradual decline in substitution frequency.

Based on the Shannon entropy analysis and substitution frequency statistics calculated using R language, residues 91, 101, and 232 of the VP2 protein exhibit significant hypervariability, with substitution frequencies markedly higher than those of other sites. Substitutions occurred 291, 677, and 71 times at these sites, respectively (Supplementary Table S3). The A91S substitution emerged around 2000 and has continuously increased, reaching 34.3% during 2020–2025, showing a clear accumulation trend (Figures 6A,B). In contrast, the frequency of A91T decreased from 7.1 to 0.17%, indicating that residue 91 is under positive selection favoring serine. The I101T substitution has remained between 50 and 75% over time (Figures 6A,C), showing little fluctuation and suggesting that this site is in a state of selective equilibrium. The V232I substitution has shown a gradual decline, with its frequency decreasing from 28.1% in the 1990s to 4.2% during 2020–2025 (Figures 6A,D). Notably, a novel substitution, V232G, appeared at this site during 2020–2025, although its frequency remains low, representing a newly emerging amino acid type at this position. Against the backdrop of the continuous decline of V232I, this surface-exposed residue may influence antigenicity and immune recognition due to changes in its physicochemical properties, warranting continued attention.

### 3.6 Regional distribution and substitution types of high-frequency sites

**Figure 7:**
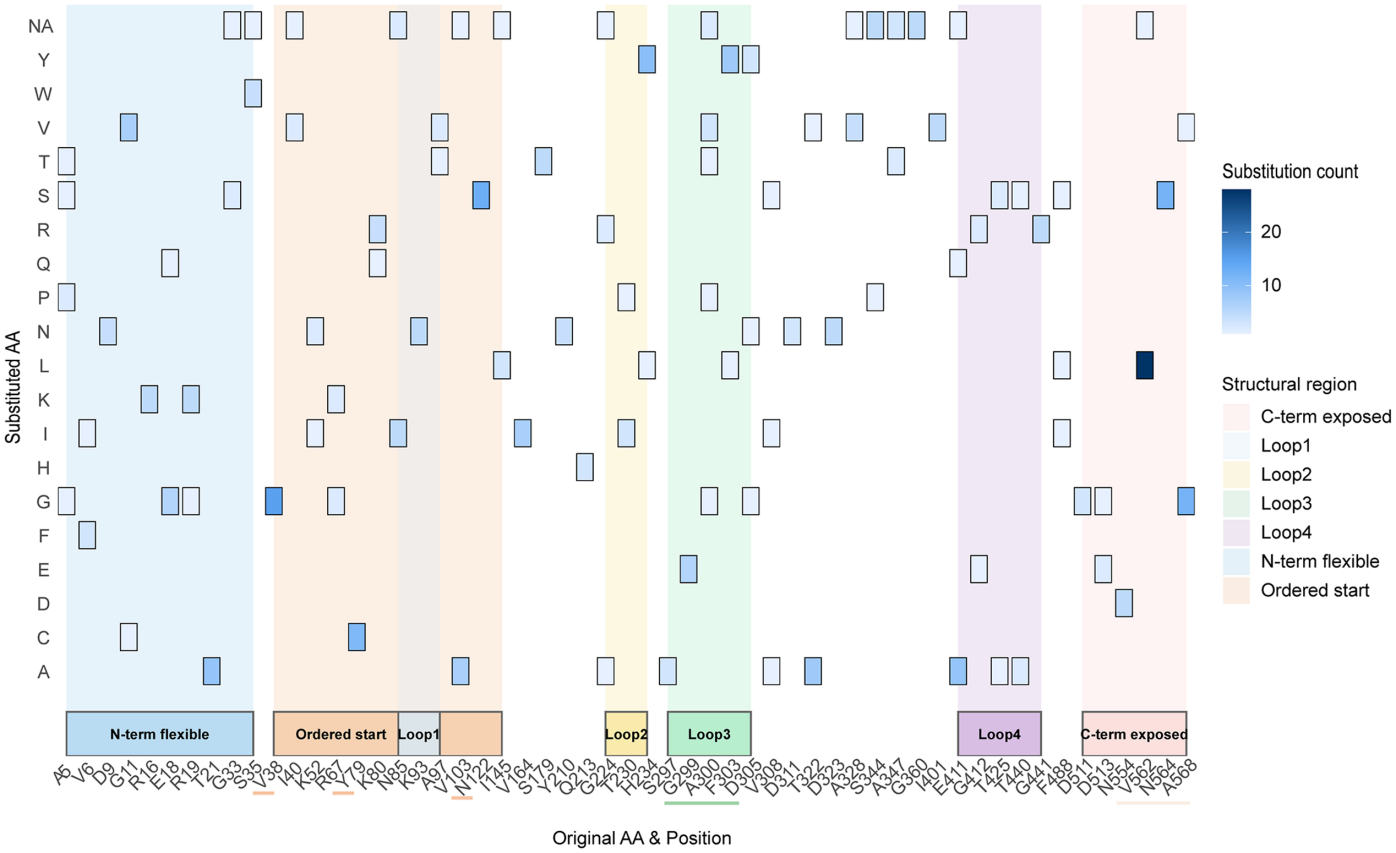
Heatmap of amino acid substitutions in key FPV VP2 residues. The vertical axis represents the “residue–original amino acid,” and the horizontal axis represents the substituted amino acid. Color intensity indicates substitution frequency, with darker colors representing higher frequencies. Known high-frequency residues A91S, I101T, and residue 232 were excluded from the statistics, and only sites with substitution counts ≥ 3 are displayed. The heatmap illustrates the cumulative substitutions across structural regions of VP2, including the N-terminal, loop regions, and C-terminal surface-exposed areas.

Based on the high-variability regions defined by the previous entropy plot, this study further calculated the substitution frequencies and types of all amino acid residues in 708 FPV VP2 sequences (Figure 7; Supplementary Table S4). The C-terminal hypervariable region showed the highest substitution density, with V562L occurring 28 times, and N564S and A568G/A568V each detected 12 times, indicating evident accumulation. This region is located in a surface-exposed loop, serving as a major hotspot for amino acid substitutions. The N-terminal and central loop regions also exhibited a number of substitutions, such as V38G (15 times), N122S (13 times), and Y79C (11 times), mainly located in exposed loop areas or near the fivefold axis channel, reflecting a multi-regional synchronous variation pattern. Additionally, the Loop 3 region on the VP2 surface (residues 299–305) showed high substitution accumulation, with over 20 total events involving at least 4 amino acid sites and 7 different substitution types (e.g., A300V/T/P, F303Y, S304P, D305N/Y). These findings indicate that substitutions in the VP2 hypervariable regions are concentrated in surface-exposed loops and outward-facing areas.

### 3.7 Analysis of substitutions in seven laboratory-isolated strains

**Figure 8:**
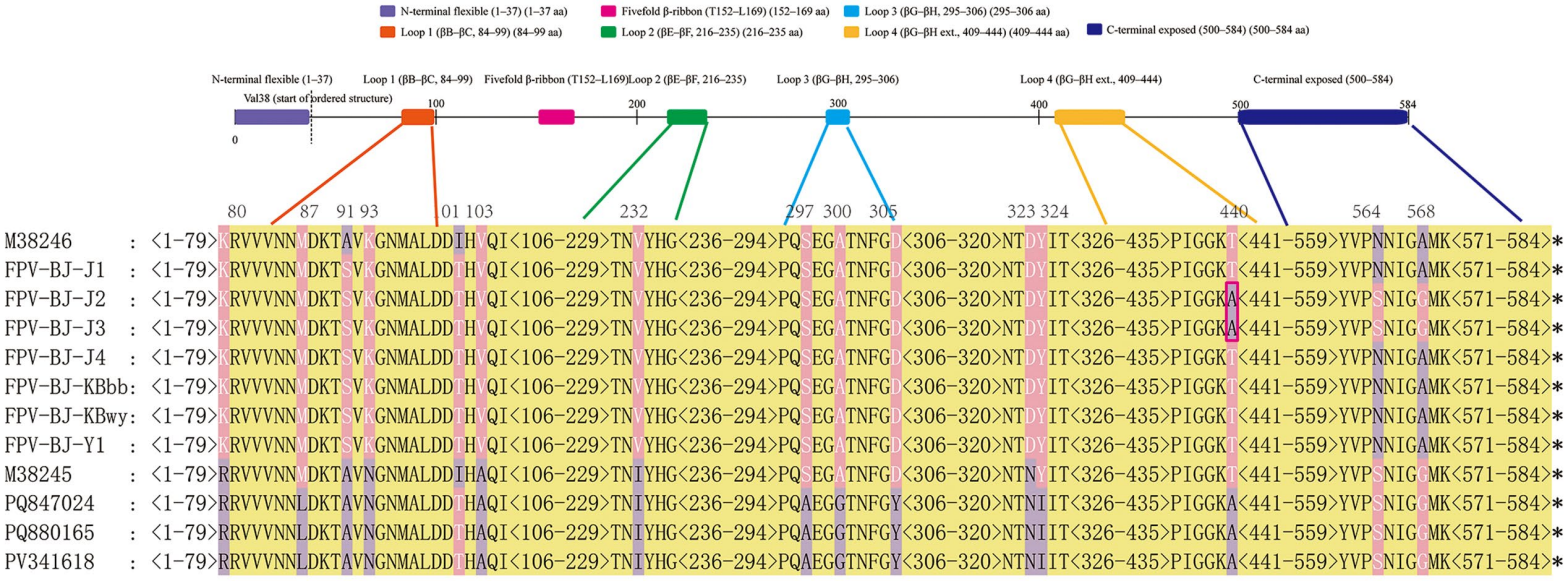
Amino acid comparison at key VP2 residues between FPV isolates from Beijing and the reference strain M38246. The alignment includes seven FPV isolates (FPV-BJ-J1 to FPV-BJ-J4, FPV-BJ-KBbb, FPV-BJ-KBwy, FPV-BJ-Y1), the FPV reference strain M38246, and several recent CPV variants. Structural and antigenic regions—βA–βB loop, loops 1–4, and the C-terminal region—are indicated by colored boxes. Conserved residues are shaded in yellow, and variable sites at positions 80, 91, 101, 232, 440, 564, and 568 are highlighted.

Compared with FPV sequence (GenBank accession no. M38246), all seven VP2 sequences isolated in this study carried the A91S and I101T substitutions (Figure 8). Notably, the FPV-BJ-J2 and FPV-BJ-J3 strains exhibited the T440A substitution, which was identified in FPV for the first time, along with the N564S and A568G substitutions, further exhibiting a CPV-characteristic substitution pattern. Previous studies on CPV have demonstrated that site T440 is located in a critical surface-exposed region of the capsid and may be associated with host adaptation or immune evasion. A91S and I101T are situated within major antigenic epitopes and may further influence viral antigenicity. Apart from these variations, the key sites related to host range determination (80 K, 93 K, 103 V, and 323D) remained conserved across all seven isolates, consistent with the FPV strain M38246.

## 4 Discussion

In recent years, the VP2 gene of FPV has undergone continuous accumulation of amino acid substitutions. These substitutions may not only modulate viral pathogenicity (26), but also compromise the cross-protective efficacy of existing vaccines by altering antigenic conformations (27), thereby attracting considerable attention. A systematic analysis of the occurrence patterns and evolutionary dynamics of VP2 mutations is therefore of great theoretical and practical significance for vaccine strain optimization and the prevention and control of parvoviral diseases.

Among the numerous substitutions identified within the FPV VP2 gene, the A91S substitution has drawn particular attention. Molecular structural prediction suggests that this substitution may extend the random coil region of the VP2 protein from residues 92–95 to 91–95, resulting in a conformational shift of Loop 1 (aa 89–91) from an *α*-helix to a disordered coil. Such an alteration may consequently modify the surface electrostatic potential and reduce the binding efficiency of neutralizing antibodies (14, 28). Since 2017, FPV variants harboring the A91S substitution have formed an independent epidemic branch within domestic cat in China and have subsequently disseminated to multiple countries. These variants frequently co-occur with characteristic substitutions in NS1 and VP1, suggesting that they may have evolved along an independent evolutionary trajectory. The I101T substitution is also predicted to influence the surface charge distribution, potentially enhancing receptor-binding affinity. Animal challenge experiments demonstrated that FPV strains carrying both A91S and I101T substitutions remain pathogenic in cats (29, 30). Our analysis revealed that the frequency of the A91S substitution has increased annually since 2017 (Figures 6A,B), suggesting ongoing positive selection pressure. The I101T substitution maintained a consistently high frequency throughout the study period (Figures 6A,C), suggesting that both mutations confer evolutionary advantages, while the dynamic changes at different sites may reflect varying host selection pressures.

Compared with the recombinant FPV/MT270571 (BJ-A240) strain isolated in Beijing in 2019 (31), which is a putative recombinant strain of CPV-2c and FPV, the two FPV isolates obtained in 2025 in this study also carried the N564S and A568G substitutions. While in this study a novel T440A substitution was identified for the first time (Figure 8). The site 440 is located at the tip of Loop 4 within the VP2 pentameric structural domain (aa 410–440). Previous studies have shown that this region is exposed on the capsid surface and may be involved in viral entry into host cells, while also representing an immunodominant site readily recognized by neutralizing antibodies (32, 33).

This study revealed that amino acid substitutions in the FPV VP2 protein were predominantly concentrated in the surface-exposed C terminus, the hypervariable loop regions, and the N-terminal loop domain (Figure 5). These regions are generally involved in receptor binding and the formation of antigenic epitopes. Notably, the G299E and A300P substitutions located within the Loop 3 region have been reported to alter the interaction between VP2 and host receptors, potentially contributing to an expanded host range (34). In conjunction with the coordinated variations observed across multiple regions in this study—particularly the C-terminal hypervariable substitutions V562L, N564S, and A568G/V—our findings suggest that FPV may be undergoing continuous adaptation to host environments or immune pressures through surface-exposed substitutions. Moreover, as these key residues are located adjacent to major antigenic clusters, their structural alterations may exert potential effects on the cross-protective efficacy of current vaccines (26, 27).

The currently Feline Viral Rhinotracheitis, Calicivirus, and Panleukopenia trivalent vaccine still provides basic immune protection for kittens as early as 6 weeks of age (35). However, its cross-protective efficacy and duration of immunity against emerging FPV variants remain inadequately evaluated. Serological investigations have revealed that, even after complete immunization, a subset of domestic cats exhibit neutralizing antibody titers below the protective threshold (36–38), suggesting that existing vaccines may not fully cover the antigenic spectrum of newly evolved FPV strains. Structural modeling and molecular prediction analyses indicate that substitutions at A91S, I101T, and site 440 within the VP2 protein may alter surface electrostatic potentials and the conformations of antigenic epitopes, thereby compromising the binding efficiency of neutralizing antibodies. These findings imply that such substitutions may play pivotal roles in FPV immune evasion and host adaptation. Further investigations into their molecular mechanisms and immunological consequences are warranted to assess the potential impact of these substitutions on the efficacy of current vaccines. In addition, the present study is mainly based on structural and evolutionary analyses of VP2 (not at the whole-genome level), and related inferences have not been validated through neutralization assays or vaccine challenge experiments; the functional consequences of these substitutions remain to be confirmed in future studies.

## 5 Conclusion

In this study, seven FPV strains were successfully isolated and identified from diarrheic cat samples collected from pet hospitals in Beijing. Their VP2 gene sequences were subjected to systematic genetic analysis and entropy-based characteristic evaluation. The results revealed that the hypervariable regions of the VP2 gene were mainly concentrated at nt 111–411, nt 477–1,038, and nt 1,500–1752. Notably, to our knowledge, the T440A substitution was detected in FPV for the first time, which is located on the viral capsid surface, an important region for host immune recognition (based on the structural position of the VP2 protein and by analogy with previously reported CPV sites). However, further studies combining serum neutralization assays are needed to evaluate its impact. Meanwhile, the A91S substitution, corresponding to the peak entropy value, has shown a significant increase in frequency over the past decade, representing the fastest-increasing amino acid substitution; the I101T substitution remains high-frequency but relatively stable, whereas substitution at site 232 have decreased year by year. Taken together, this study reveals the distribution of hypervariable regions and temporal evolution patterns of key substitution sites in the FPV VP2 protein, providing new molecular insights into FPV evolutionary dynamics. Notably, the effectiveness of vaccines against these strains requires further evaluation.

## Data Availability

The datasets presented in this study can be found in online repositories. The FPV VP2 gene sequences obtained in this study have been submitted to the GenBank database and can be accessed using the following accession numbers: FPV-BJ-J1 (GenBank accession no. PX496583), FPV-BJ-J2 (GenBank accession no. PX496584), FPV-BJ-J3 (GenBank accession no. PX496585), FPV-BJ-J4 (GenBank accession no. PX496586), FPV-BJ-Y1 (GenBank accession no. PX496587), FPV-BJ-KBwy (GenBank accession no. PX496588), and FPV-BJ-KBbb (GenBank accession no. PX496589).
